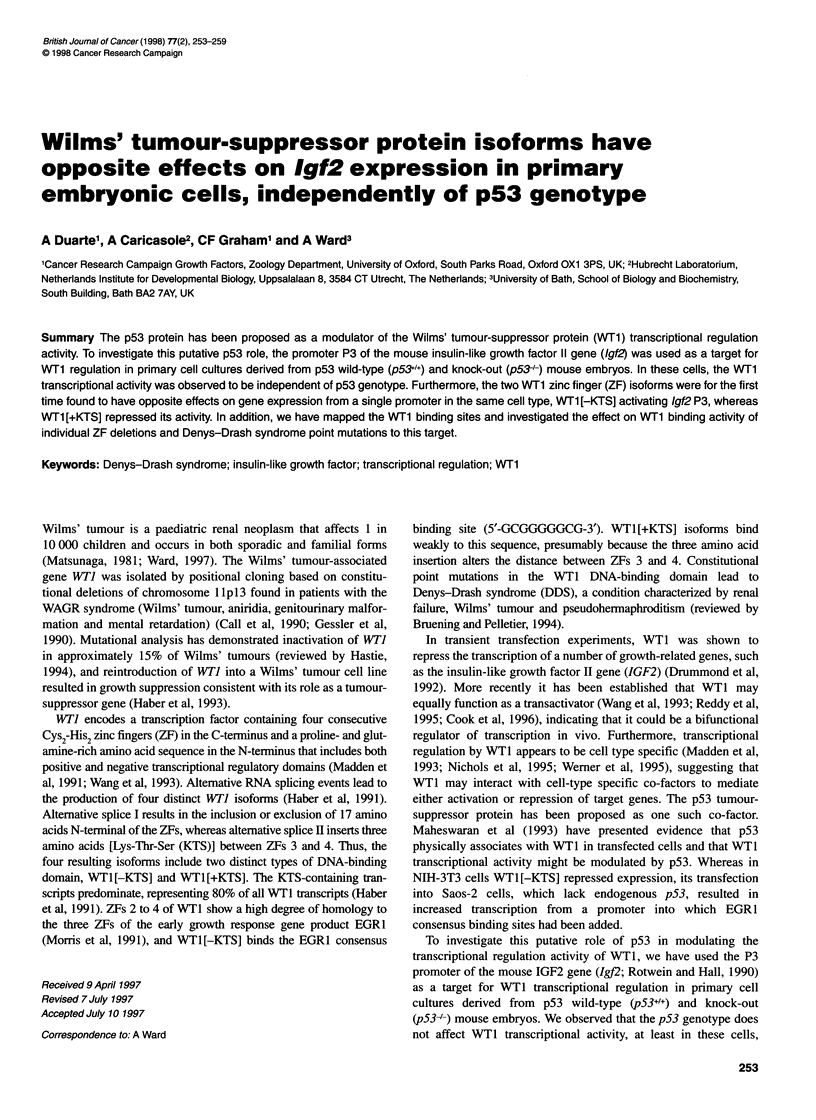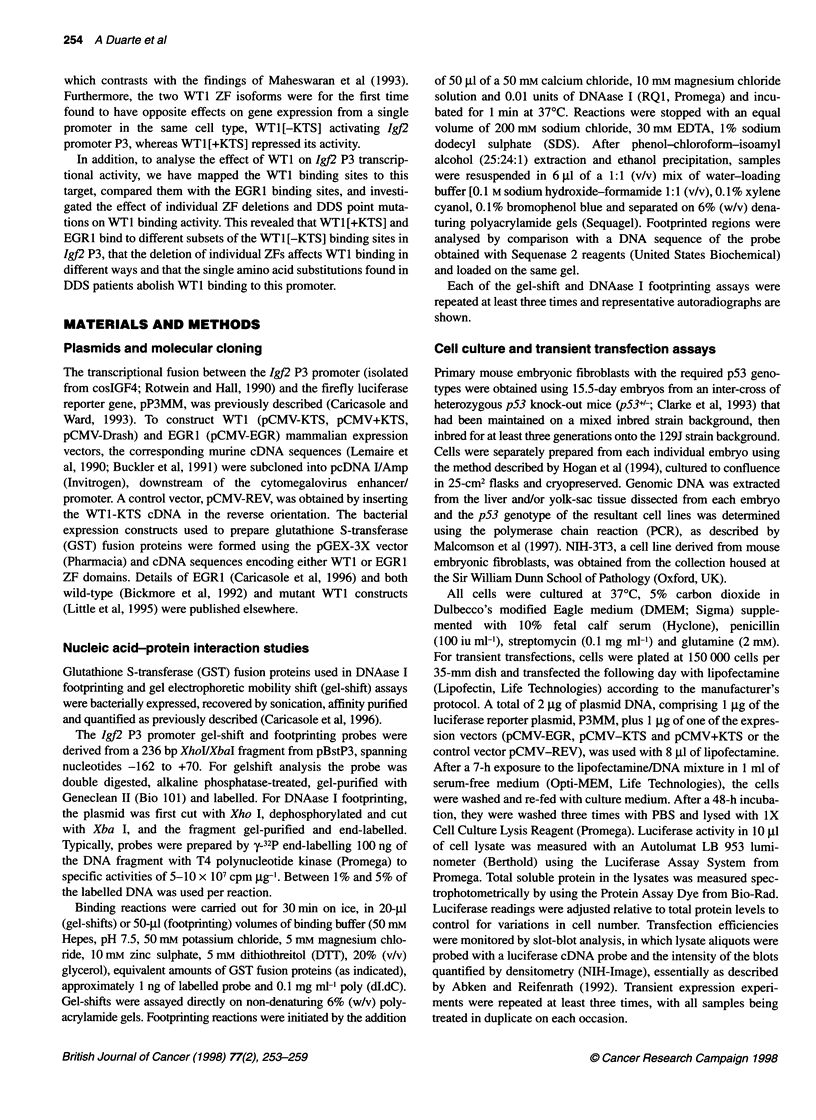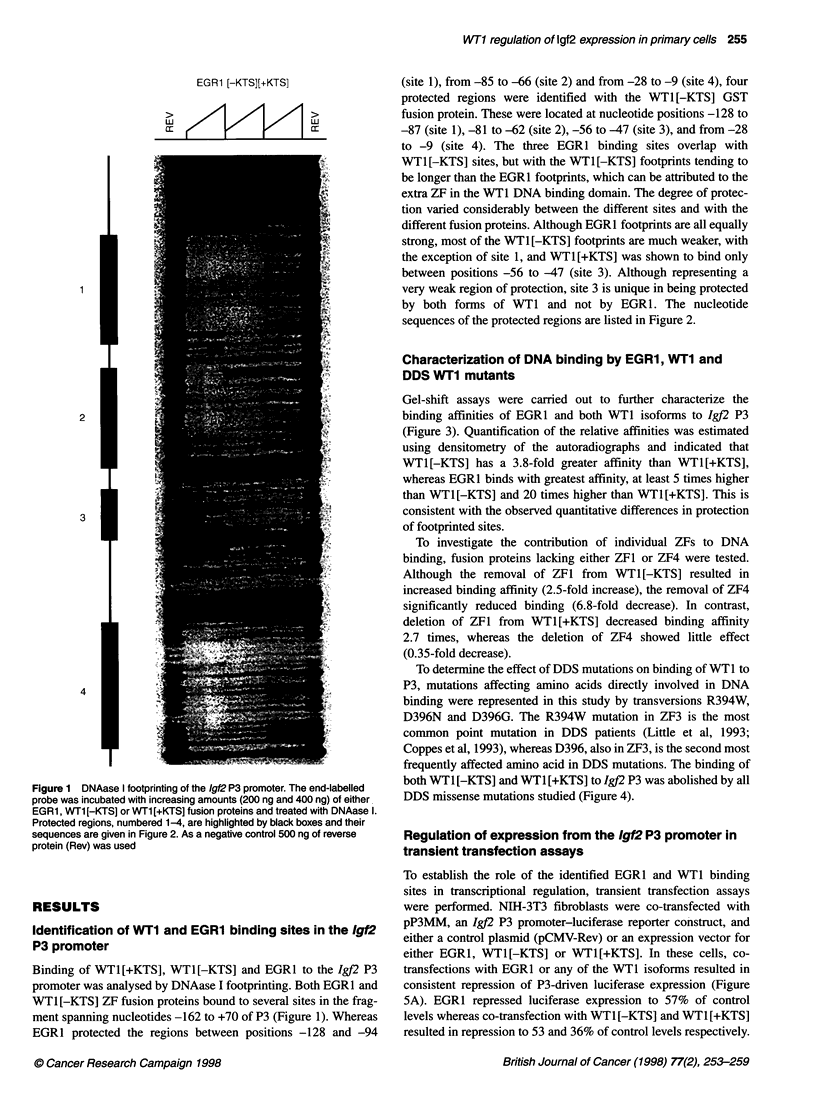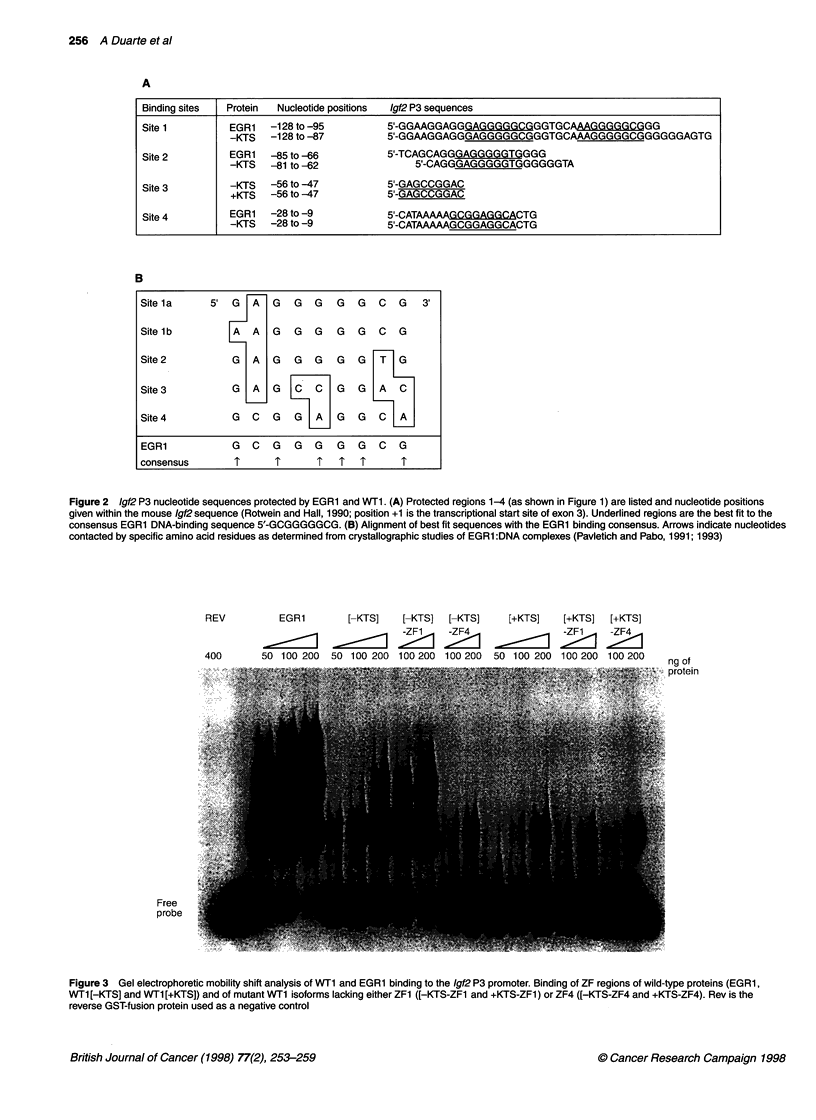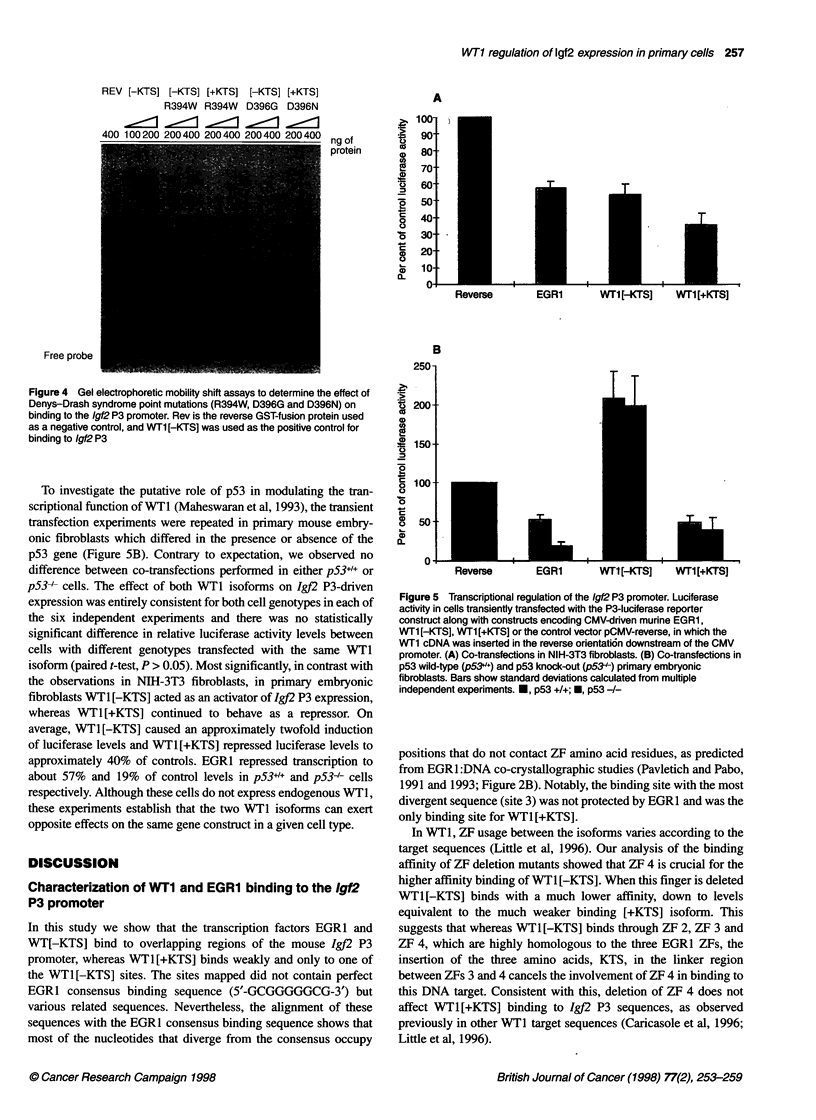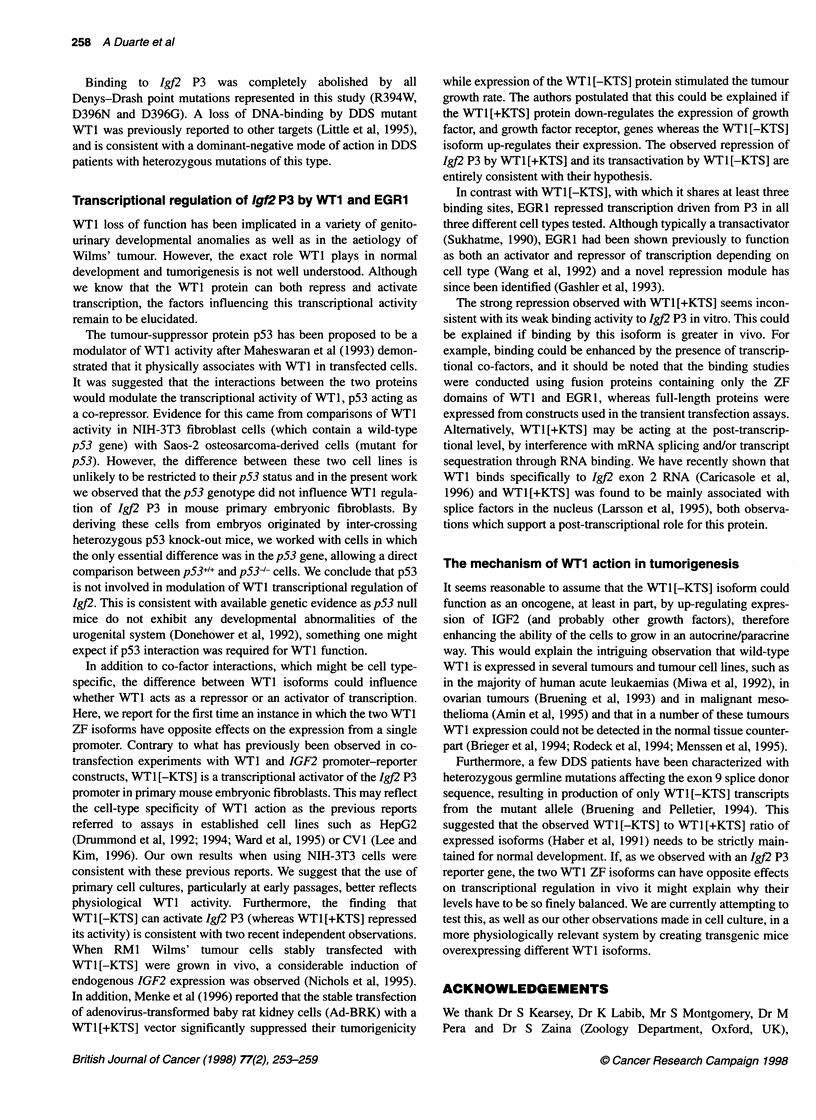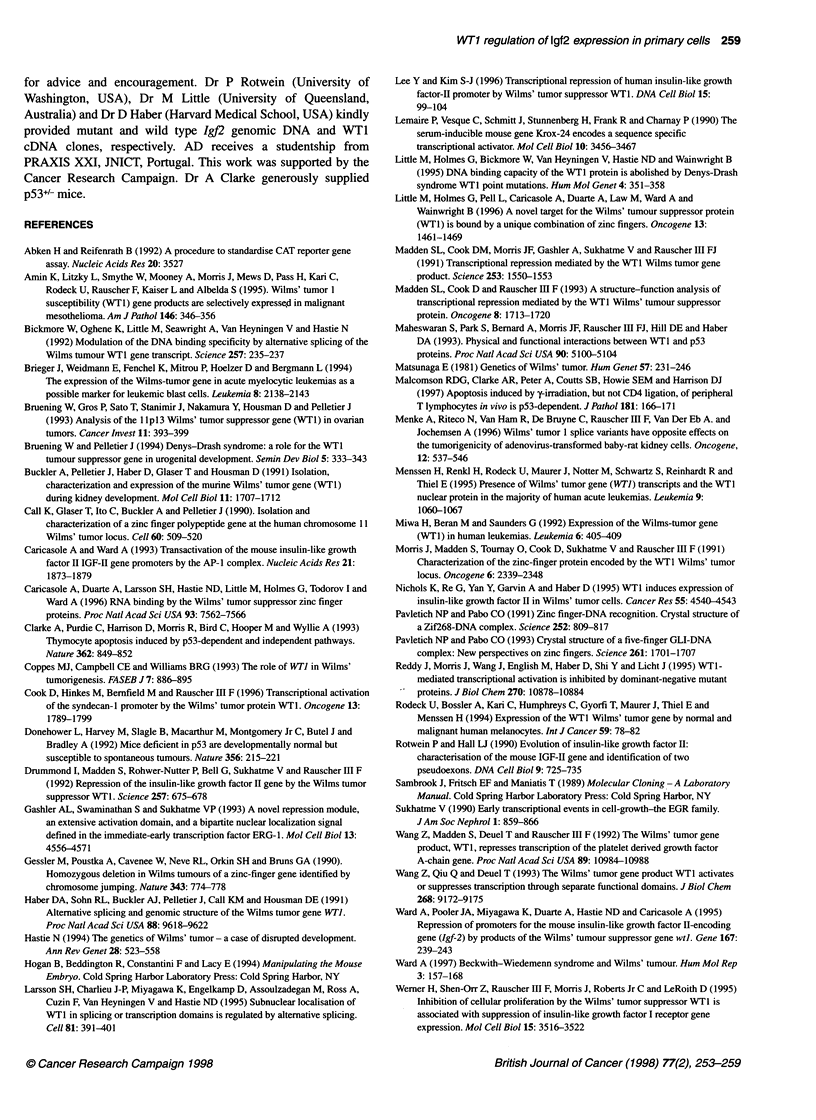# Wilms' tumour-suppressor protein isoforms have opposite effects on Igf2 expression in primary embryonic cells, independently of p53 genotype.

**DOI:** 10.1038/bjc.1998.41

**Published:** 1998

**Authors:** A. Duarte, A. Caricasole, C. F. Graham, A. Ward

**Affiliations:** Cancer Research Campaign Growth Factors, Zoology Department, University of Oxford, UK.

## Abstract

**Images:**


British Journal of Cancer (1998) 77(2), 253–259
© 1998 Cancer Research Campaign

### Wilms' tumour-suppressor protein isoforms have opposite effects on *Igf2* expression in primary embryonic cells, independently of p53 genotype

**A Duarte[1], A Caricasole[2], CF Graham[1] and A Ward[3]**

[1]Cancer Research Campaign Growth Factors, Zoology Department, University of Oxford, South Parks Road, Oxford OX1 3PS, UK; [2]Hubrecht Laboratorium, Netherlands Institute for Developmental Biology, Uppsalalaan 8, 3584 CT Utrecht, The Netherlands; [3]University of Bath, School of Biology and Biochemistry, South Building, Bath BA2 7AY, UK

**Summary** The p53 protein has been proposed as a modulator of the Wilms' tumour-suppressor protein (WT1) transcriptional regulation activity. To investigate this putative p53 role, the promoter P3 of the mouse insulin-like growth factor II gene (*Igf2*) was used as a target for WT1 regulation in primary cell cultures derived from p53 wild-type (*p53*$^{+/+}$) and knock-out (*p53*$^{-/-}$) mouse embryos. In these cells, the WT1 transcriptional activity was observed to be independent of p53 genotype. Furthermore, the two WT1 zinc finger (ZF) isoforms were for the first time found to have opposite effects on gene expression from a single promoter in the same cell type, WT1[–KTS] activating *Igf2* P3, whereas WT1[+KTS] repressed its activity. In addition, we have mapped the WT1 binding sites and investigated the effect on WT1 binding activity of individual ZF deletions and Denys–Drash syndrome point mutations to this target.

**Keywords:** Denys–Drash syndrome; insulin-like growth factor; transcriptional regulation; WT1

Wilms' tumour is a paediatric renal neoplasm that affects 1 in 10 000 children and occurs in both sporadic and familial forms (Matsunaga, 1981; Ward, 1997). The Wilms' tumour-associated gene *WT1* was isolated by positional cloning based on constitutional deletions of chromosome 11p13 found in patients with the WAGR syndrome (Wilms' tumour, aniridia, genitourinary malformation and mental retardation) (Call et al, 1990; Gessler et al, 1990). Mutational analysis has demonstrated inactivation of *WT1* in approximately 15% of Wilms' tumours (reviewed by Hastie, 1994), and reintroduction of *WT1* into a Wilms' tumour cell line resulted in growth suppression consistent with its role as a tumour-suppressor gene (Haber et al, 1993).

*WT1* encodes a transcription factor containing four consecutive $Cys_2$-$His_2$ zinc fingers (ZF) in the C-terminus and a proline- and glutamine-rich amino acid sequence in the N-terminus that includes both positive and negative transcriptional regulatory domains (Madden et al, 1991; Wang et al, 1993). Alternative RNA splicing events lead to the production of four distinct *WT1* isoforms (Haber et al, 1991). Alternative splice I results in the inclusion or exclusion of 17 amino acids N-terminal of the ZFs, whereas alternative splice II inserts three amino acids [Lys-Thr-Ser (KTS)] between ZFs 3 and 4. Thus, the four resulting isoforms include two distinct types of DNA-binding domain, WT1[–KTS] and WT1[+KTS]. The KTS-containing transcripts predominate, representing 80% of all WT1 transcripts (Haber et al, 1991). ZFs 2 to 4 of WT1 show a high degree of homology to the three ZFs of the early growth response gene product EGR1 (Morris et al, 1991), and WT1[–KTS] binds the EGR1 consensus binding site (5′-GCGGGGGCG-3′). WT1[+KTS] isoforms bind weakly to this sequence, presumably because the three amino acid insertion alters the distance between ZFs 3 and 4. Constitutional point mutations in the WT1 DNA-binding domain lead to Denys–Drash syndrome (DDS), a condition characterized by renal failure, Wilms' tumour and pseudohermaphroditism (reviewed by Bruening and Pelletier, 1994).

In transient transfection experiments, WT1 was shown to repress the transcription of a number of growth-related genes, such as the insulin-like growth factor II gene (*IGF2*) (Drummond et al, 1992). More recently it has been established that WT1 may equally function as a transactivator (Wang et al, 1993; Reddy et al, 1995; Cook et al, 1996), indicating that it could be a bifunctional regulator of transcription in vivo. Furthermore, transcriptional regulation by WT1 appears to be cell type specific (Madden et al, 1993; Nichols et al, 1995; Werner et al, 1995), suggesting that WT1 may interact with cell-type specific co-factors to mediate either activation or repression of target genes. The p53 tumour-suppressor protein has been proposed as one such co-factor. Maheswaran et al (1993) have presented evidence that p53 physically associates with WT1 in transfected cells and that WT1 transcriptional activity might be modulated by p53. Whereas in NIH-3T3 cells WT1[–KTS] repressed expression, its transfection into Saos-2 cells, which lack endogenous *p53*, resulted in increased transcription from a promoter into which EGR1 consensus binding sites had been added.

To investigate this putative role of p53 in modulating the transcriptional regulation activity of WT1, we have used the P3 promoter of the mouse IGF2 gene (*Igf2*; Rotwein and Hall, 1990) as a target for WT1 transcriptional regulation in primary cell cultures derived from p53 wild-type (*p53*$^{+/+}$) and knock-out (*p53*$^{-/-}$) mouse embryos. We observed that the *p53* genotype does not affect WT1 transcriptional activity, at least in these cells,

*Received 9 April 1997*
*Revised 7 July 1997*
*Accepted July 10 1997*

*Correspondence to:* A Ward which contrasts with the findings of Maheswaran et al (1993). Furthermore, the two WT1 ZF isoforms were for the first time found to have opposite effects on gene expression from a single promoter in the same cell type, WT1[–KTS] activating *Igf2* promoter P3, whereas WT1[+KTS] repressed its activity.

In addition, to analyse the effect of WT1 on *Igf2* P3 transcriptional activity, we have mapped the WT1 binding sites to this target, compared them with the EGR1 binding sites, and investigated the effect of individual ZF deletions and DDS point mutations on WT1 binding activity. This revealed that WT1[+KTS] and EGR1 bind to different subsets of the WT1[–KTS] binding sites in *Igf2* P3, that the deletion of individual ZFs affects WT1 binding in different ways and that the single amino acid substitutions found in DDS patients abolish WT1 binding to this promoter.

### MATERIALS AND METHODS

### Plasmids and molecular cloning

The transcriptional fusion between the *Igf2* P3 promoter (isolated from cosIGF4; Rotwein and Hall, 1990) and the firefly luciferase reporter gene, pP3MM, was previously described (Caricasole and Ward, 1993). To construct WT1 (pCMV-KTS, pCMV+KTS, pCMV-Drash) and EGR1 (pCMV-EGR) mammalian expression vectors, the corresponding murine cDNA sequences (Lemaire et al, 1990; Buckler et al, 1991) were subcloned into pcDNA I/Amp (Invitrogen), downstream of the cytomegalovirus enhancer/promoter. A control vector, pCMV-REV, was obtained by inserting the WT1-KTS cDNA in the reverse orientation. The bacterial expression constructs used to prepare glutathione S-transferase (GST) fusion proteins were formed using the pGEX-3X vector (Pharmacia) and cDNA sequences encoding either WT1 or EGR1 ZF domains. Details of EGR1 (Caricasole et al, 1996) and both wild-type (Bickmore et al, 1992) and mutant WT1 constructs (Little et al, 1995) were published elsewhere.

### Nucleic acid–protein interaction studies

Glutathione S-transferase (GST) fusion proteins used in DNAase I footprinting and gel electrophoretic mobility shift (gel-shift) assays were bacterially expressed, recovered by sonication, affinity purified and quantified as previously described (Caricasole et al, 1996).

The *Igf2* P3 promoter gel-shift and footprinting probes were derived from a 236 bp *Xho*I/*Xba*I fragment from pBstP3, spanning nucleotides –162 to +70. For gelshift analysis the probe was double digested, alkaline phosphatase-treated, gel-purified with Geneclean II (Bio 101) and labelled. For DNAase I footprinting, the plasmid was first cut with *Xho* I, dephosphorylated and cut with *Xba* I, and the fragment gel-purified and end-labelled. Typically, probes were prepared by $\gamma$-$^{32}$P end-labelling 100 ng of the DNA fragment with T4 polynucleotide kinase (Promega) to specific activities of $5–10 \times 10^7$ cpm $\mu g^{-1}$. Between 1% and 5% of the labelled DNA was used per reaction.

Binding reactions were carried out for 30 min on ice, in 20-μl (gel-shifts) or 50-μl (footprinting) volumes of binding buffer (50 mM Hepes, pH 7.5, 50 mM potassium chloride, 5 mM magnesium chloride, 10 mM zinc sulphate, 5 mM dithiothreitol (DTT), 20% (v/v) glycerol), equivalent amounts of GST fusion proteins (as indicated), approximately 1 ng of labelled probe and 0.1 mg ml$^{-1}$ poly (dI.dC). Gel-shifts were assayed directly on non-denaturing 6% (w/v) polyacrylamide gels. Footprinting reactions were initiated by the addition of 50 μl of a 50 mM calcium chloride, 10 mM magnesium chloride solution and 0.01 units of DNAase I (RQ1, Promega) and incubated for 1 min at 37°C. Reactions were stopped with an equal volume of 200 mM sodium chloride, 30 mM EDTA, 1% sodium dodecyl sulphate (SDS). After phenol–chloroform–isoamyl alcohol (25:24:1) extraction and ethanol precipitation, samples were resuspended in 6 μl of a 1:1 (v/v) mix of water–loading buffer [0.1 M sodium hydroxide–formamide 1:1 (v/v), 0.1% xylene cyanol, 0.1% bromophenol blue and separated on 6% (w/v) denaturing polyacrylamide gels (Sequagel). Footprinted regions were analysed by comparison with a DNA sequence of the probe obtained with Sequenase 2 reagents (United States Biochemical) and loaded on the same gel.

Each of the gel-shift and DNAase I footprinting assays were repeated at least three times and representative autoradiographs are shown.

### Cell culture and transient transfection assays

Primary mouse embryonic fibroblasts with the required p53 genotypes were obtained using 15.5-day embryos from an inter-cross of heterozygous *p53* knock-out mice (*p53*$^{+/-}$; Clarke et al, 1993) that had been maintained on a mixed inbred strain background, then inbred for at least three generations onto the 129J strain background. Cells were separately prepared from each individual embryo using the method described by Hogan et al (1994), cultured to confluence in 25-cm$^2$ flasks and cryopreserved. Genomic DNA was extracted from the liver and/or yolk-sac tissue dissected from each embryo and the *p53* genotype of the resultant cell lines was determined using the polymerase chain reaction (PCR), as described by Malcomson et al (1997). NIH-3T3, a cell line derived from mouse embryonic fibroblasts, was obtained from the collection housed at the Sir William Dunn School of Pathology (Oxford, UK).

All cells were cultured at 37°C, 5% carbon dioxide in Dulbecco's modified Eagle medium (DMEM; Sigma) supplemented with 10% fetal calf serum (Hyclone), penicillin (100 iu ml$^{-1}$), streptomycin (0.1 mg ml$^{-1}$) and glutamine (2 mM). For transient transfections, cells were plated at 150 000 cells per 35-mm dish and transfected the following day with lipofectamine (Lipofectin, Life Technologies) according to the manufacturer's protocol. A total of 2 μg of plasmid DNA, comprising 1 μg of the luciferase reporter plasmid, P3MM, plus 1 μg of one of the expression vectors (pCMV-EGR, pCMV–KTS and pCMV+KTS or the control vector pCMV–REV), was used with 8 μl of lipofectamine. After a 7-h exposure to the lipofectamine/DNA mixture in 1 ml of serum-free medium (Opti-MEM, Life Technologies), the cells were washed and re-fed with culture medium. After a 48-h incubation, they were washed three times with PBS and lysed with 1X Cell Culture Lysis Reagent (Promega). Luciferase activity in 10 μl of cell lysate was measured with an Autolumat LB 953 luminometer (Berthold) using the Luciferase Assay System from Promega. Total soluble protein in the lysates was measured spectrophotometrically by using the Protein Assay Dye from Bio-Rad. Luciferase readings were adjusted relative to total protein levels to control for variations in cell number. Transfection efficiencies were monitored by slot-blot analysis, in which lysate aliquots were probed with a luciferase cDNA probe and the intensity of the blots quantified by densitometry (NIH-Image), essentially as described by Abken and Reifenrath (1992). Transient expression experiments were repeated at least three times, with all samples being treated in duplicate on each occasion.

© Cancer Research Campaign 1998

**Figure 1** DNAase I footprinting of the *Igf2* P3 promoter. The end-labelled probe was incubated with increasing amounts (200 ng and 400 ng) of either EGR1, WT1[–KTS] or WT1[+KTS] fusion proteins and treated with DNAase I. Protected regions, numbered 1–4, are highlighted by black boxes and their sequences are given in Figure 2. As a negative control 500 ng of reverse protein (Rev) was used

### RESULTS

### Identification of WT1 and EGR1 binding sites in the *Igf2* P3 promoter

Binding of WT1[+KTS], WT1[–KTS] and EGR1 to the *Igf2* P3 promoter was analysed by DNAase I footprinting. Both EGR1 and WT1[–KTS] ZF fusion proteins bound to several sites in the fragment spanning nucleotides –162 to +70 of P3 (Figure 1). Whereas EGR1 protected the regions between positions –128 and –94 (site 1), from –85 to –66 (site 2) and from –28 to –9 (site 4), four protected regions were identified with the WT1[–KTS] GST fusion protein. These were located at nucleotide positions –128 to –87 (site 1), –81 to –62 (site 2), –56 to –47 (site 3), and from –28 to –9 (site 4). The three EGR1 binding sites overlap with WT1[–KTS] sites, but with the WT1[–KTS] footprints tending to be longer than the EGR1 footprints, which can be attributed to the extra ZF in the WT1 DNA binding domain. The degree of protection varied considerably between the different sites and with the different fusion proteins. Although EGR1 footprints are all equally strong, most of the WT1[–KTS] footprints are much weaker, with the exception of site 1, and WT1[+KTS] was shown to bind only between positions –56 to –47 (site 3). Although representing a very weak region of protection, site 3 is unique in being protected by both forms of WT1 and not by EGR1. The nucleotide sequences of the protected regions are listed in Figure 2.

### Characterization of DNA binding by EGR1, WT1 and DDS WT1 mutants

Gel-shift assays were carried out to further characterize the binding affinities of EGR1 and both WT1 isoforms to *Igf2* P3 (Figure 3). Quantification of the relative affinities was estimated using densitometry of the autoradiographs and indicated that WT1[–KTS] has a 3.8-fold greater affinity than WT1[+KTS], whereas EGR1 binds with greatest affinity, at least 5 times higher than WT1[–KTS] and 20 times higher than WT1[+KTS]. This is consistent with the observed quantitative differences in protection of footprinted sites.

To investigate the contribution of individual ZFs to DNA binding, fusion proteins lacking either ZF1 or ZF4 were tested. Although the removal of ZF1 from WT1[–KTS] resulted in increased binding affinity (2.5-fold increase), the removal of ZF4 significantly reduced binding (6.8-fold decrease). In contrast, deletion of ZF1 from WT1[+KTS] decreased binding affinity 2.7 times, whereas the deletion of ZF4 showed little effect (0.35-fold decrease).

To determine the effect of DDS mutations on binding of WT1 to P3, mutations affecting amino acids directly involved in DNA binding were represented in this study by transversions R394W, D396N and D396G. The R394W mutation in ZF3 is the most common point mutation in DDS patients (Little et al, 1993; Coppes et al, 1993), whereas D396, also in ZF3, is the second most frequently affected amino acid in DDS mutations. The binding of both WT1[–KTS] and WT1[+KTS] to *Igf2* P3 was abolished by all DDS missense mutations studied (Figure 4).

### Regulation of expression from the *Igf2* P3 promoter in transient transfection assays

To establish the role of the identified EGR1 and WT1 binding sites in transcriptional regulation, transient transfection assays were performed. NIH-3T3 fibroblasts were co-transfected with pP3MM, an *Igf2* P3 promoter–luciferase reporter construct, and either a control plasmid (pCMV-Rev) or an expression vector for either EGR1, WT1[–KTS] or WT1[+KTS]. In these cells, co-transfections with EGR1 or any of the WT1 isoforms resulted in consistent repression of P3-driven luciferase expression (Figure 5A). EGR1 repressed luciferase expression to 57% of control levels whereas co-transfection with WT1[–KTS] and WT1[+KTS] resulted in repression to 53 and 36% of control levels respectively.

© Cancer Research Campaign 1998 

**A**

| Binding sites | Protein | Nucleotide positions | *Igf2* P3 sequences |
|---|---|---|---|
| Site 1 | EGR1<br>–KTS | –128 to –95<br>–128 to –87 | 5'-GGAAGGAGGGAGGGGGCGGGTGCAAAGGGGGCGGG<br>5'-GGAAGGAGGGAGGGGGCGGGTGCAAAGGGGGCGGGGGGAGTG |
| Site 2 | EGR1<br>–KTS | –85 to –66<br>–81 to –62 | 5'-TCAGCAGGGAGGGGGTGGGG<br>5'-CAGGGAGGGGGTGGGGGTA |
| Site 3 | –KTS<br>+KTS | –56 to –47<br>–56 to –47 | 5'-GAGCCGGAC<br>5'-GAGCCGGAC |
| Site 4 | EGR1<br>–KTS | –28 to –9<br>–28 to –9 | 5'-CATAAAAAGCGGAGGCACTG<br>5'-CATAAAAAGCGGAGGCACTG |

**B**

| | | | | | | | | | | | |
|---|---|---|---|---|---|---|---|---|---|---|---|
| Site 1a | 5' | G | A | G | G | G | G | G | C | G | 3' |
| Site 1b | | A | A | G | G | G | G | G | C | G | |
| Site 2 | | G | A | G | G | G | G | G | T | G | |
| Site 3 | | G | A | G | C | C | G | G | A | C | |
| Site 4 | | G | C | G | G | A | G | G | C | A | |
| EGR1 | | G | C | G | G | G | G | G | C | G | |
| consensus | | ↑ | | ↑ | | ↑ | ↑ | ↑ | | ↑ | |

**Figure 2** *Igf2* P3 nucleotide sequences protected by EGR1 and WT1. (**A**) Protected regions 1–4 (as shown in Figure 1) are listed and nucleotide positions given within the mouse *Igf2* sequence (Rotwein and Hall, 1990; position +1 is the transcriptional start site of exon 3). Underlined regions are the best fit to the consensus EGR1 DNA-binding sequence 5′-GCGGGGGCG. (**B**) Alignment of best fit sequences with the EGR1 binding consensus. Arrows indicate nucleotides contacted by specific amino acid residues as determined from crystallographic studies of EGR1:DNA complexes (Pavletich and Pabo, 1991; 1993)

**Figure 3** Gel electrophoretic mobility shift analysis of WT1 and EGR1 binding to the *Igf2* P3 promoter. Binding of ZF regions of wild-type proteins (EGR1, WT1[–KTS] and WT1[+KTS]) and of mutant WT1 isoforms lacking either ZF1 ([–KTS-ZF1 and +KTS-ZF1) or ZF4 ([–KTS-ZF4 and +KTS-ZF4). Rev is the reverse GST-fusion protein used as a negative control

© Cancer Research Campaign 1998

**Figure 4** Gel electrophoretic mobility shift assays to determine the effect of Denys–Drash syndrome point mutations (R394W, D396G and D396N) on binding to the *Igf2* P3 promoter. Rev is the reverse GST-fusion protein used as a negative control, and WT1[–KTS] was used as the positive control for binding to *Igf2* P3

**Figure 5** Transcriptional regulation of the *Igf2* P3 promoter. Luciferase activity in cells transiently transfected with the P3-luciferase reporter construct along with constructs encoding CMV-driven murine EGR1, WT1[–KTS], WT1[+KTS] or the control vector pCMV-reverse, in which the WT1 cDNA was inserted in the reverse orientation downstream of the CMV promoter. (**A**) Co-transfections in NIH-3T3 fibroblasts. (**B**) Co-transfections in p53 wild-type (*p53*$^{+/+}$) and p53 knock-out (*p53*$^{-/-}$) primary embryonic fibroblasts. Bars show standard deviations calculated from multiple independent experiments. ■, p53 +/+; ■, p53 –/–

To investigate the putative role of p53 in modulating the transcriptional function of WT1 (Maheswaran et al, 1993), the transient transfection experiments were repeated in primary mouse embryonic fibroblasts which differed in the presence or absence of the p53 gene (Figure 5B). Contrary to expectation, we observed no difference between co-transfections performed in either *p53*$^{+/+}$ or *p53*$^{-/-}$ cells. The effect of both WT1 isoforms on *Igf2* P3-driven expression was entirely consistent for both cell genotypes in each of the six independent experiments and there was no statistically significant difference in relative luciferase activity levels between cells with different genotypes transfected with the same WT1 isoform (paired *t*-test, $P > 0.05$). Most significantly, in contrast with the observations in NIH-3T3 fibroblasts, in primary embryonic fibroblasts WT1[–KTS] acted as an activator of *Igf2* P3 expression, whereas WT1[+KTS] continued to behave as a repressor. On average, WT1[–KTS] caused an approximately twofold induction of luciferase levels and WT1[+KTS] repressed luciferase levels to approximately 40% of controls. EGR1 repressed transcription to about 57% and 19% of control levels in *p53*$^{+/+}$ and *p53*$^{-/-}$ cells respectively. Although these cells do not express endogenous WT1, these experiments establish that the two WT1 isoforms can exert opposite effects on the same gene construct in a given cell type.

### DISCUSSION

### Characterization of WT1 and EGR1 binding to the *Igf2* P3 promoter

In this study we show that the transcription factors EGR1 and WT[–KTS] bind to overlapping regions of the mouse *Igf2* P3 promoter, whereas WT1[+KTS] binds weakly and only to one of the WT1[–KTS] sites. The sites mapped did not contain perfect EGR1 consensus binding sequence (5′-GCGGGGGCG-3′) but various related sequences. Nevertheless, the alignment of these sequences with the EGR1 consensus binding sequence shows that most of the nucleotides that diverge from the consensus occupy positions that do not contact ZF amino acid residues, as predicted from EGR1:DNA co-crystallographic studies (Pavletich and Pabo, 1991 and 1993; Figure 2B). Notably, the binding site with the most divergent sequence (site 3) was not protected by EGR1 and was the only binding site for WT1[+KTS].

In WT1, ZF usage between the isoforms varies according to the target sequences (Little et al, 1996). Our analysis of the binding affinity of ZF deletion mutants showed that ZF 4 is crucial for the higher affinity binding of WT1[–KTS]. When this finger is deleted WT1[–KTS] binds with a much lower affinity, down to levels equivalent to the much weaker binding [+KTS] isoform. This suggests that whereas WT1[–KTS] binds through ZF 2, ZF 3 and ZF 4, which are highly homologous to the three EGR1 ZFs, the insertion of the three amino acids, KTS, in the linker region between ZFs 3 and 4 cancels the involvement of ZF 4 in binding to this DNA target. Consistent with this, deletion of ZF 4 does not affect WT1[+KTS] binding to *Igf2* P3 sequences, as observed previously in other WT1 target sequences (Caricasole et al, 1996; Little et al, 1996).

© Cancer Research Campaign 1998

Binding to *Igf2* P3 was completely abolished by all Denys–Drash point mutations represented in this study (R394W, D396N and D396G). A loss of DNA-binding by DDS mutant WT1 was previously reported to other targets (Little et al, 1995), and is consistent with a dominant-negative mode of action in DDS patients with heterozygous mutations of this type.

### Transcriptional regulation of *Igf2* P3 by WT1 and EGR1

WT1 loss of function has been implicated in a variety of genitourinary developmental anomalies as well as in the aetiology of Wilms' tumour. However, the exact role WT1 plays in normal development and tumorigenesis is not well understood. Although we know that the WT1 protein can both repress and activate transcription, the factors influencing this transcriptional activity remain to be elucidated.

The tumour-suppressor protein p53 has been proposed to be a modulator of WT1 activity after Maheswaran et al (1993) demonstrated that it physically associates with WT1 in transfected cells. It was suggested that the interactions between the two proteins would modulate the transcriptional activity of WT1, p53 acting as a co-repressor. Evidence for this came from comparisons of WT1 activity in NIH-3T3 fibroblast cells (which contain a wild-type *p53* gene) with Saos-2 osteosarcoma-derived cells (mutant for *p53*). However, the difference between these two cell lines is unlikely to be restricted to their *p53* status and in the present work we observed that the *p53* genotype did not influence WT1 regulation of *Igf2* P3 in mouse primary embryonic fibroblasts. By deriving these cells from embryos originated by inter-crossing heterozygous p53 knock-out mice, we worked with cells in which the only essential difference was in the *p53* gene, allowing a direct comparison between $p53^{+/+}$ and $p53^{-/-}$ cells. We conclude that p53 is not involved in modulation of WT1 transcriptional regulation of *Igf2*. This is consistent with available genetic evidence as *p53* null mice do not exhibit any developmental abnormalities of the urogenital system (Donehower et al, 1992), something one might expect if p53 interaction was required for WT1 function.

In addition to co-factor interactions, which might be cell type-specific, the difference between WT1 isoforms could influence whether WT1 acts as a repressor or an activator of transcription. Here, we report for the first time an instance in which the two WT1 ZF isoforms have opposite effects on the expression from a single promoter. Contrary to what has previously been observed in co-transfection experiments with WT1 and *IGF2* promoter–reporter constructs, WT1[–KTS] is a transcriptional activator of the *Igf2* P3 promoter in primary mouse embryonic fibroblasts. This may reflect the cell-type specificity of WT1 action as the previous reports referred to assays in established cell lines such as HepG2 (Drummond et al, 1992; 1994; Ward et al, 1995) or CV1 (Lee and Kim, 1996). Our own results when using NIH-3T3 cells were consistent with these previous reports. We suggest that the use of primary cell cultures, particularly at early passages, better reflects physiological WT1 activity. Furthermore, the finding that WT1[–KTS] can activate *Igf2* P3 (whereas WT1[+KTS] repressed its activity) is consistent with two recent independent observations. When RM1 Wilms' tumour cells stably transfected with WT1[–KTS] were grown in vivo, a considerable induction of endogenous *IGF2* expression was observed (Nichols et al, 1995). In addition, Menke et al (1996) reported that the stable transfection of adenovirus-transformed baby rat kidney cells (Ad-BRK) with a WT1[+KTS] vector significantly suppressed their tumorigenicity while expression of the WT1[–KTS] protein stimulated the tumour growth rate. The authors postulated that this could be explained if the WT1[+KTS] protein down-regulates the expression of growth factor, and growth factor receptor, genes whereas the WT1[–KTS] isoform up-regulates their expression. The observed repression of *Igf2* P3 by WT1[+KTS] and its transactivation by WT1[–KTS] are entirely consistent with their hypothesis.

In contrast with WT1[–KTS], with which it shares at least three binding sites, EGR1 repressed transcription driven from P3 in all three different cell types tested. Although typically a transactivator (Sukhatme, 1990), EGR1 had been shown previously to function as both an activator and repressor of transcription depending on cell type (Wang et al, 1992) and a novel repression module has since been identified (Gashler et al, 1993).

The strong repression observed with WT1[+KTS] seems inconsistent with its weak binding activity to *Igf2* P3 in vitro. This could be explained if binding by this isoform is greater in vivo. For example, binding could be enhanced by the presence of transcriptional co-factors, and it should be noted that the binding studies were conducted using fusion proteins containing only the ZF domains of WT1 and EGR1, whereas full-length proteins were expressed from constructs used in the transient transfection assays. Alternatively, WT1[+KTS] may be acting at the post-transcriptional level, by interference with mRNA splicing and/or transcript sequestration through RNA binding. We have recently shown that WT1 binds specifically to *Igf2* exon 2 RNA (Caricasole et al, 1996) and WT1[+KTS] was found to be mainly associated with splice factors in the nucleus (Larsson et al, 1995), both observations which support a post-transcriptional role for this protein.

### The mechanism of WT1 action in tumorigenesis

It seems reasonable to assume that the WT1[–KTS] isoform could function as an oncogene, at least in part, by up-regulating expression of IGF2 (and probably other growth factors), therefore enhancing the ability of the cells to grow in an autocrine/paracrine way. This would explain the intriguing observation that wild-type WT1 is expressed in several tumours and tumour cell lines, such as in the majority of human acute leukaemias (Miwa et al, 1992), in ovarian tumours (Bruening et al, 1993) and in malignant mesothelioma (Amin et al, 1995) and that in a number of these tumours WT1 expression could not be detected in the normal tissue counterpart (Brieger et al, 1994; Rodeck et al, 1994; Menssen et al, 1995).

Furthermore, a few DDS patients have been characterized with heterozygous germline mutations affecting the exon 9 splice donor sequence, resulting in production of only WT1[–KTS] transcripts from the mutant allele (Bruening and Pelletier, 1994). This suggested that the observed WT1[–KTS] to WT1[+KTS] ratio of expressed isoforms (Haber et al, 1991) needs to be strictly maintained for normal development. If, as we observed with an *Igf2* P3 reporter gene, the two WT1 ZF isoforms can have opposite effects on transcriptional regulation in vivo it might explain why their levels have to be so finely balanced. We are currently attempting to test this, as well as our other observations made in cell culture, in a more physiologically relevant system by creating transgenic mice overexpressing different WT1 isoforms.

### ACKNOWLEDGEMENTS

We thank Dr S Kearsey, Dr K Labib, Mr S Montgomery, Dr M Pera and Dr S Zaina (Zoology Department, Oxford, UK),

© Cancer Research Campaign 1998

for advice and encouragement. Dr P Rotwein (University of Washington, USA), Dr M Little (University of Queensland, Australia) and Dr D Haber (Harvard Medical School, USA) kindly provided mutant and wild type *Igf2* genomic DNA and WT1 cDNA clones, respectively. AD receives a studentship from PRAXIS XXI, JNICT, Portugal. This work was supported by the Cancer Research Campaign. Dr A Clarke generously supplied $p53^{+/-}$ mice.

### REFERENCES

Abken H and Reifenrath B (1992) A procedure to standardise CAT reporter gene assay. *Nucleic Acids Res* **20**: 3527

Amin K, Litzky L, Smythe W, Mooney A, Morris J, Mews D, Pass H, Kari C, Rodeck U, Rauscher F, Kaiser L and Albelda S (1995). Wilms' tumor 1 susceptibility (WT1) gene products are selectively expressed in malignant mesothelioma. *Am J Pathol* **146**: 346–356

Bickmore W, Oghene K, Little M, Seawright A, Van Heyningen V and Hastie N (1992) Modulation of the DNA binding specificity by alternative splicing of the Wilms tumour WT1 gene transcript. *Science* **257**: 235–237

Brieger J, Weidmann E, Fenchel K, Mitrou P, Hoelzer D and Bergmann L (1994) The expression of the Wilms-tumor gene in acute myelocytic leukemias as a possible marker for leukemic blast cells. *Leukemia* **8**: 2138–2143

Bruening W, Gros P, Sato T, Stanimir J, Nakamura Y, Housman D and Pelletier J (1993) Analysis of the 11p13 Wilms' tumor suppressor gene (WT1) in ovarian tumors. *Cancer Invest* **11**: 393–399

Bruening W and Pelletier J (1994) Denys–Drash syndrome: a role for the WT1 tumour suppressor gene in urogenital development. *Semin Dev Biol* **5**: 333–343

Buckler A, Pelletier J, Haber D, Glaser T and Housman D (1991) Isolation, characterization and expression of the murine Wilms' tumor gene (WT1) during kidney development. *Mol Cell Biol* **11**: 1707–1712

Call K, Glaser T, Ito C, Buckler A and Pelletier J (1990). Isolation and characterization of a zinc finger polypeptide gene at the human chromosome 11 Wilms' tumor locus. *Cell* **60**: 509–520

Caricasole A and Ward A (1993) Transactivation of the mouse insulin-like growth factor II IGF-II gene promoters by the AP-1 complex. *Nucleic Acids Res* **21**: 1873–1879

Caricasole A, Duarte A, Larsson SH, Hastie ND, Little M, Holmes G, Todorov I and Ward A (1996) RNA binding by the Wilms' tumor suppressor zinc finger proteins. *Proc Natl Acad Sci USA* **93**: 7562–7566

Clarke A, Purdie C, Harrison D, Morris R, Bird C, Hooper M and Wyllie A (1993) Thymocyte apoptosis induced by p53-dependent and independent pathways. *Nature* **362**: 849–852

Coppes MJ, Campbell CE and Williams BRG (1993) The role of *WT1* in Wilms' tumorigenesis. *FASEB J* **7**: 886–895

Cook D, Hinkes M, Bernfield M and Rauscher III F (1996) Transcriptional activation of the syndecan-1 promoter by the Wilms' tumor protein WT1. *Oncogene* **13**: 1789–1799

Donehower L, Harvey M, Slagle B, Macarthur M, Montgomery Jr C, Butel J and Bradley A (1992) Mice deficient in p53 are developmentally normal but susceptible to spontaneous tumours. *Nature* **356**: 215–221

Drummond I, Madden S, Rohwer-Nutter P, Bell G, Sukhatme V and Rauscher III F (1992) Repression of the insulin-like growth factor II gene by the Wilms tumor suppressor WT1. *Science* **257**: 675–678

Gashler AL, Swaminathan S and Sukhatme VP (1993) A novel repression module, an extensive activation domain, and a bipartite nuclear localization signal defined in the immediate-early transcription factor ERG-1. *Mol Cell Biol* **13**: 4556–4571

Gessler M, Poustka A, Cavenee W, Neve RL, Orkin SH and Bruns GA (1990). Homozygous deletion in Wilms tumours of a zinc-finger gene identified by chromosome jumping. *Nature* **343**: 774–778

Haber DA, Sohn RL, Buckler AJ, Pelletier J, Call KM and Housman DE (1991) Alternative splicing and genomic structure of the Wilms tumor gene *WT1*. *Proc Natl Acad Sci USA* **88**: 9618–9622

Hastie N (1994) The genetics of Wilms' tumor – a case of disrupted development. *Ann Rev Genet* **28**: 523–558

Hogan B, Beddington R, Constantini F and Lacy E (1994) *Manipulating the Mouse Embryo*. Cold Spring Harbor Laboratory Press: Cold Spring Harbor, NY

Larsson SH, Charlieu J-P, Miyagawa K, Engelkamp D, Assoulzadegan M, Ross A, Cuzin F, Van Heyningen V and Hastie ND (1995) Subnuclear localisation of WT1 in splicing or transcription domains is regulated by alternative splicing. *Cell* **81**: 391–401

Lee Y and Kim S-J (1996) Transcriptional repression of human insulin-like growth factor-II promoter by Wilms' tumor suppressor WT1. *DNA Cell Biol* **15**: 99–104

Lemaire P, Vesque C, Schmitt J, Stunnenberg H, Frank R and Charnay P (1990) The serum-inducible mouse gene Krox-24 encodes a sequence specific transcriptional activator. *Mol Cell Biol* **10**: 3456–3467

Little M, Holmes G, Bickmore W, Van Heyningen V, Hastie ND and Wainwright B (1995) DNA binding capacity of the WT1 protein is abolished by Denys-Drash syndrome WT1 point mutations. *Hum Mol Genet* **4**: 351–358

Little M, Holmes G, Pell L, Caricasole A, Duarte A, Law M, Ward A and Wainwright B (1996) A novel target for the Wilms' tumour suppressor protein (WT1) is bound by a unique combination of zinc fingers. *Oncogene* **13**: 1461–1469

Madden SL, Cook DM, Morris JF, Gashler A, Sukhatme V and Rauscher III FJ (1991) Transcriptional repression mediated by the WT1 Wilms tumor gene product. *Science* **253**: 1550–1553

Madden SL, Cook D and Rauscher III F (1993) A structure–function analysis of transcriptional repression mediated by the WT1 Wilms' tumour suppressor protein. *Oncogene* **8**: 1713–1720

Maheswaran S, Park S, Bernard A, Morris JF, Rauscher III FJ, Hill DE and Haber DA (1993). Physical and functional interactions between WT1 and p53 proteins. *Proc Natl Acad Sci USA* **90**: 5100–5104

Matsunaga E (1981) Genetics of Wilms' tumor. *Hum Genet* **57**: 231–246

Malcomson RDG, Clarke AR, Peter A, Coutts SB, Howie SEM and Harrison DJ (1997) Apoptosis induced by γ-irradiation, but not CD4 ligation, of peripheral T lymphocytes *in vivo* is p53-dependent. *J Pathol* **181**: 166–171

Menke A, Riteco N, Van Ham R, De Bruyne C, Rauscher III F, Van Der Eb A. and Jochemsen A (1996) Wilms' tumor 1 splice variants have opposite effects on the tumorigenicity of adenovirus-transformed baby-rat kidney cells. *Oncogene*, **12**: 537–546

Menssen H, Renkl H, Rodeck U, Maurer J, Notter M, Schwartz S, Reinhardt R and Thiel E (1995) Presence of Wilms' tumor gene (*WT1*) transcripts and the WT1 nuclear protein in the majority of human acute leukemias. *Leukemia* **9**: 1060–1067

Miwa H, Beran M and Saunders G (1992) Expression of the Wilms-tumor gene (WT1) in human leukemias. *Leukemia* **6**: 405–409

Morris J, Madden S, Tournay O, Cook D, Sukhatme V and Rauscher III F (1991) Characterization of the zinc-finger protein encoded by the WT1 Wilms' tumor locus. *Oncogene* **6**: 2339–2348

Nichols K, Re G, Yan Y, Garvin A and Haber D (1995) WT1 induces expression of insulin-like growth factor II in Wilms' tumor cells. *Cancer Res* **55**: 4540–4543

Pavletich NP and Pabo CO (1991) Zinc finger-DNA recognition. Crystal structure of a Zif268-DNA complex. *Science* **252**: 809–817

Pavletich NP and Pabo CO (1993) Crystal structure of a five-finger GLI-DNA complex: New perspectives on zinc fingers. *Science* **261**: 1701–1707

Reddy J, Morris J, Wang J, English M, Haber D, Shi Y and Licht J (1995) WT1-mediated transcriptional activation is inhibited by dominant-negative mutant proteins. *J Biol Chem* **270**: 10878–10884

Rodeck U, Bossler A, Kari C, Humphreys C, Gyorfi T, Maurer J, Thiel E and Menssen H (1994) Expression of the WT1 Wilms' tumor gene by normal and malignant human melanocytes. *Int J Cancer* **59**: 78–82

Rotwein P and Hall LJ (1990) Evolution of insulin-like growth factor II: characterisation of the mouse IGF-II gene and identification of two pseudoexons. *DNA Cell Biol* **9**: 725–735

Sambrook J, Fritsch EF and Maniatis T (1989) *Molecular Cloning – A Laboratory Manual*. Cold Spring Harbor Laboratory Press: Cold Spring Harbor, NY

Sukhatme V (1990) Early transcriptional events in cell-growth–the EGR family. *J Am Soc Nephrol* **1**: 859–866

Wang Z, Madden S, Deuel T and Rauscher III F (1992) The Wilms' tumor gene product, WT1, represses transcription of the platelet derived growth factor A-chain gene. *Proc Natl Acad Sci USA* **89**: 10984–10988

Wang Z, Qiu Q and Deuel T (1993) The Wilms' tumor gene product WT1 activates or suppresses transcription through separate functional domains. *J Biol Chem* **268**: 9172–9175

Ward A, Pooler JA, Miyagawa K, Duarte A, Hastie ND and Caricasole A (1995) Repression of promoters for the mouse insulin-like growth factor II-encoding gene (*Igf-2*) by products of the Wilms' tumour suppressor gene *wt1*. *Gene* **167**: 239–243

Ward A (1997) Beckwith–Wiedemenn syndrome and Wilms' tumour. *Hum Mol Rep* **3**: 157–168

Werner H, Shen-Orr Z, Rauscher III F, Morris J, Roberts Jr C and LeRoith D (1995) Inhibition of cellular proliferation by the Wilms' tumor suppressor WT1 is associated with suppression of insulin-like growth factor I receptor gene expression. *Mol Cell Biol* **15**: 3516–3522

© Cancer Research Campaign 1998